# Omphalocele Secondary to Spontaneous Rupture of Allantoic Cyst in the Third Trimester of Pregnancy

**DOI:** 10.1155/2021/6940685

**Published:** 2021-09-18

**Authors:** Giuliana Orlandi, Paolo Toscano, Lavinia Di Meglio, Letizia Di Meglio, Aniello Di Meglio

**Affiliations:** ^1^Department of Neuroscience, Reproductive Sciences and Dentistry, School of Medicine, University of Naples Federico II, Naples, Italy; ^2^Diagnostica Ecografica e Prenatale di A. Di Meglio, Naples, Italy

## Abstract

**Objective:**

We report the first case in which the onset of omphalocele was after the spontaneous rupture of an allantoic cyst. We hypothesize a causal link between the spontaneous rupture of the cyst and the herniation of the viscera. *Case Presentation*. A 36-year-old woman was diagnosed with an allantoic cyst during the first trimester. The allantoic cyst underwent spontaneous rupture during the 32nd week of gestation, and an omphalocele developed secondary to the cyst's rupture. Two days after birth, the peritoneum covering intestinal loops broke spontaneously and the newborn underwent successful urgent surgery.

**Conclusions:**

This case may suggest that the relative benignity of the allantoid cysts may recommend a close ultrasound follow-up in order to identify the onset of any complications, as a late third trimester onset of omphalocele. Prenatal diagnosis of such complications may allow multidisciplinary management of the pregnancy with planned cesarean section, prenatal pediatric surgery consultation, and neonatal surgery.

## 1. Introduction

In the early stages of embryogenesis, the bladder's dome communicates with the allantoid, a structure located inside the umbilical cord, through the urachus. Patent urachus in the prenatal period has an estimated incidence of 3 cases out of 1,000,000, three times more frequent in males [1]. These anomalies must be included in the differential diagnosis of cystic formations of the umbilical cord [2]. During the first trimester, the prevalence of umbilical cord cysts ranges from 0.4 to 3.4% [3]. These must be differentiated into true cysts, such as the allantoic cyst, the omphalomesenteric cyst, amniotic inclusion cysts and some vascular malformations, and pseudocysts, which are essential areas of degeneration of Wharton's jelly [4]. The clinical significance of these anomalies is still uncertain, although the association with chromosomal and anatomical anomalies is described in the literature. The most frequent chromosomal anomalies are trisomies 13 and 18 [5], while anatomical ones are omphalocele and gastroschisis [6]. In particular, allantoic cysts and omphalocele are described as two coexisting anomalies in the literature [7].

Here, to the best of our knowledge, we report the first case of omphalocele after spontaneous rupture of the allantoic cyst, highlighting the need for a close follow-up of the cystic pathologies of the umbilical cord in order to exclude the onset of this complication.

## 2. Case Report

**Figure 1:**
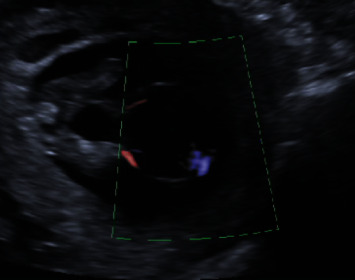
Allantoic cyst during ultrasound examination at the 13th week of gestation. Note its position within the umbilical cord and adjacent to the abdominal wall. It is avascular, in communication with the bladder and embraced by the two umbilical arteries.

A 36-year-old woman at her third pregnancy was referred to our third level prenatal ultrasound diagnostic center to undergo the first trimester screening exam during the 13th week of gestation. Fetal anatomy was regular, except for the presence of an anomalous umbilical cord cyst. It had a maximum size of 18 mm and regular margins and was adjacent to the abdominal wall. This cyst was avascular, had no internal septa or vegetations, and was within the umbilical cord, embraced by the two umbilical arteries, in communication with the bladder. Its size varied during the ultrasound examination, which lasted several hours, configuring the diagnostic suspicion of an allantoic cyst with patent urachus (Figure 1).

**Figure 2:**
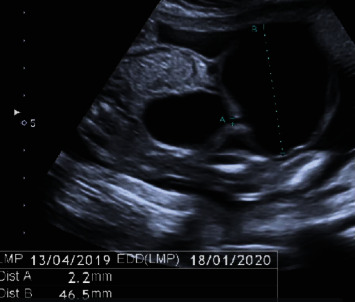
Same allantoic cyst during the second trimester scan examination at the 22nd week of gestation. Appreciate the increased size and the communication with the bladder.

**Figure 3:**
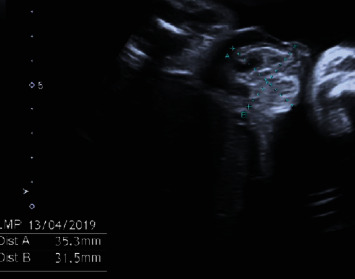
In the ultrasound examination performed at the 32nd week of gestation, the allantoic cyst was no longer visible and replaced by an omphalocele of 35 × 31 millimeters, secondary to the cyst's rupture.

Amniocentesis was performed to rule out chromosomal or genomic anomalies. The traditional karyotype study and array-based Comparative Genomic Hybridization- (CGH-) array showed no alterations related to an allantoic cyst, such as trisomies 13 and 18. Subsequently, the patient underwent a second trimester screening exam at our center during the 21st week of gestation. The biometric values appeared overall greater than a week compared to gestational age, and the allantoic cyst showed an increased size, with a maximum diameter of 46 mm; the remaining anatomy explored by ultrasound was regular (Figure 2). In the following ultrasound check, performed during the 32nd week of gestation, the cyst and the bladder were no longer visible, due to cyst rupture, while an omphalocele of 35 × 31 millimeters appeared, consisting of loops of small intestines coated with membranes (Figure 3). The patient then underwent fetal magnetic resonance imaging, which confirmed the ultrasound findings and showed a stretching of the bladder dome through the abdominal wall defect.

**Figure 4:**
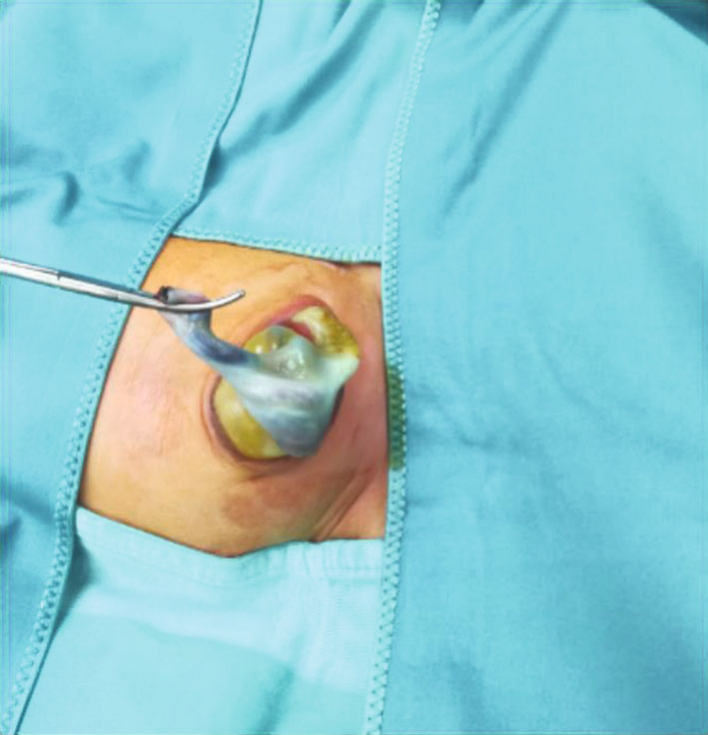
Postnatal confirmation of omphalocele which needed urgent surgical correction because of spontaneous rupture of the peritoneum.

Gestation was carried out regularly, and delivery was by elective cesarean section in a facility equipped with neonatal intensive care and pediatric surgery unit. Two days after birth, the peritoneum covering intestinal loops broke spontaneously, and the newborn underwent successful urgent surgery (Figure 4). Subsequent pathological examination found the presence of uroepithelium, confirming the prenatal diagnostic suspicion of an allantoic cyst.

## 3. Discussion

The urogenital system derives embryologically from the intermediate mesoderm; in the beginning, the excretory ducts of both apparatuses (urinary and genital) end inside a common cavity, called the cloaca [8]. Between the 4th and 7th week of development, the cloaca divides into the urogenital sinus, from which the bladder will derive, and the urorectal sinus [8]. In this period, the bladder communicates with the allantoid, blind-ended duct located in the umbilical cord [8]. After the descent of the bladder inside the pelvis, around the 16th day of development, the allantoid undergoes a physiological process of obliteration; it gives rise to a fibrous cord, the urachus, which will constitute the median bladder ligament [8]. Anomalies of this process lead to the development of umbilical cord cysts [9], diagnosable during the first trimester screening of pregnancy.

During the first trimester screening examination, a cystic anomaly of the umbilical cord could come across the sonographer. These anomalies are differentiated into true cysts, located near the abdominal wall, and pseudocysts, generally more distal and frequent [1]. True cysts include the allantoic, the omphalomesenteric, and the amniotic inclusion cysts and some vascular malformations [2]. Pseudocysts are represented by Wharton's jelly degeneration areas [2]. Furthermore, while the allantoic cyst develops in the center of the umbilical cord, leading to progressive diffuse cord edema and separation of the umbilical vessels, the pseudocysts are located in the lateral part of the cord, pushing both umbilical arteries laterally [10]. The cystic formations resulting from the persistence of the urachus and the allantoid are the urachal sinus, the urachal cyst, patent urachus, and the allantoic cyst [11]. Urachal sinus and patent urachus are malformations diagnosed in the postnatal period due to the appearance of recurrent urinary tract infections or budding of urine from the navel [12]. On the contrary, the urachal and the allantoic cysts are diagnosable during the prenatal period. The urachal cyst appears as anechoic intra-abdominal median formation, which overhangs and communicates with the bladder, embraced by the umbilical arteries [10]. Its dimensions may change during the ultrasound examination, due to bladder emptying. The urachal cyst must be considered in the differential diagnosis of the aforementioned umbilical cystic formations and with omphalocele and bladder exstrophy [10]. On the other hand, the allantoic cyst appears as a transonic formation in the context of the umbilical cord, near the abdominal wall; this formation is in continuity with the bladder through the patent urachus and is surrounded by the umbilical arteries [10].

Clinically, these anomalies are most often asymptomatic; however, they can lead to urinary tract infections and kidney reflux hydronephrosis [10].

The natural history of these cysts during the prenatal period is their progressive enlargement and, consequently, the rupture. It occurs more often between the 22nd and 32nd weeks of gestation [13].

This event leads to a free communication of the bladder with the amniotic cavity and consequently its failure of repletion, with preserved amniotic fluid [2, 10].

The prevalence of cord cysts during the first trimester screening is 3.4% [3]. The association between these cysts and chromosomopathies is still unclear. Although studies in the literature have linked them [5], some reviews have denied this association, suggesting an increased risk of chromosomopathies only in the case of multiple umbilical cysts or associated fetal abnormalities [6]. Among these, the most frequent is omphalocele, whose coexistence with allantoic anomalies during the first trimester has already been documented [7]. However, in no case, the omphalocele developed after the allantoic cyst rupture.

Here, we report the first case in which the onset of omphalocele was after the spontaneous rupture of the allantoic cyst. In particular, the diagnosis was made at 12 weeks of pregnancy. The ultrasound follow-up revealed a progressive increase in size and its subsequent rupture after the 21st week. Lastly, during the third trimester, our case was characterized by a late development of omphalocele. We hypothesize a causal link between the spontaneous rupture of the cyst and the herniation of the viscera, due to the creation of a locus minoris resistentiae of the abdominal wall, hernial gate of the omphalocele. Therefore, besides reported associations with chromosomal and anatomical abnormalities, this report highlights the necessity of a close follow-up of allantoic cysts due to the possible development of omphalocele. Early diagnosis of such complications is crucial for pregnancy management. In fact, although barring any fetal complications, preterm delivery is not recommended in neonates with omphalocele [14]; cesarean section delivery should be a more advisable choice in case of a large omphalocele because of the risks of in utero torsion of the viscera and fetal demise [15]. Moreover, early diagnosis of such complications may allow a multidisciplinary management of the pregnancy with pediatric surgery consultation and computed tomography scan at birth for the evaluation of omphalocele or any bladder lesions.

The anomalies of the urachus-allantoic system usually occur during the first trimester of pregnancy. Once the presence of associated anatomical or genetic abnormalities is excluded, the relative benignity of the allantoid cysts may recommend a close ultrasound follow-up in order to identify the onset of any complications, as a late third trimester onset of omphalocele. Prenatal diagnosis of such complications may allow multidisciplinary management of the pregnancy with planned cesarean section, prenatal pediatric surgery consultation, computed tomography scan at birth for the evaluation of omphalocele or any bladder lesions, and neonatal surgery.

## Data Availability

The data used to support the findings of this study are included within the article.
